# Incidence, risk factors, management and prevention of severe postoperative epistaxis after endoscopic endonasal transsphenoidal surgery: a single center experience

**DOI:** 10.3389/fsurg.2023.1203409

**Published:** 2023-07-26

**Authors:** Xiaohai Liu, Pengfei Wang, Mingchu Li, Ge Chen

**Affiliations:** ^1^ Department of Neurosurgery, Xuanwu Hospital Capital Medical University, Beijing, China; ^2^ Department of Neurosurgery, Hebei General Hospital, Shijiazhuang, China

**Keywords:** epistaxis, endoscopic endonasal transsphenoidal surgery, posterior nasal septal artery, electrocauterization, prevention

## Abstract

**Objective:**

Postoperative epistaxis is a very rare but severe complication after endoscopic endonasal transsphenoidal surgery (EETS) that can lead to catastrophic consequences. However, the incidence, risk factors, management and prevention of postoperative epistaxis remain unclear.

**Patients and methods:**

Consecutive patients with pituitary adenoma (PA), Rathke's cleft cyst, craniopharyngioma, or clival chordoma who received EETS in our department between September 2020 and November 2022 were retrospectively analyzed. The incidence, risk factors, management and prevention of postoperative epistaxis were investigated and analyzed.

**Results:**

A total of 557 consecutive patients who received EETS were included in this study. Eight patients (1.4%) (7 PAs and 1 Rathke's cleft cyst) experienced severe postoperative epistaxis. The size of the PAs was 9.6 mm–46.2 mm, with a median size of 22.1 mm. Epistaxis occurred 4 h to 30 days (median 14.5 days) postoperatively. Bleeding was stopped in 3 patients after nasal packing with iodoform gauze. The remaining 5 patients for whom nasal packing was insufficient were all sent to the operating room, and posterior nasal septal artery (PNSA) bleeding was identified and successfully treated with endoscopic bleeding artery electrocauterization under general anesthesia. In the EETS, all 8 patients had downward extension of the septal mucosal incision, in which 6 had intraoperative bleeding of PNSA that were cauterized by bipolar diathermy. Four patients had causative factors, including administration of antiplatelet agents, Valsalva-like manoeuvre, nose blowing and removal of nasal packing, respectively. No patients had recurrent epistaxis during the follow-up period.

**Conclusion:**

Post-EETS epistaxis is a rare but severe complication that could lead to catastrophic consequences, and one of the most common bleeding sources is the PNSA. Endoscopic bleeding artery electrocauterization under general anesthesia may be a safe, economic and effective measure for epistaxis refractory to nasal packing. Avoiding excessive downward extension of the septal mucosal incision could contribute to the prevention of postoperative epistaxis.

## Introduction

Since its development in 1992 under a minimally invasive strategy, the endoscopic endonasal transsphenoidal surgery (EETS) has been commonly used for lesions in the sellar, suprasellar or parasellar regions, including pituitary adenomas (PAs), craniopharyngiomas, Rathke's cleft cyst, meningiomas, and chordomas (1, 2). Due to the complex anatomy of the sellar region and characteristic nature of the lesions, the EETS also leads to a higher risk of complications, such as neurovascular damage, skull base repair failure and intracranial infection (3). Among the complications of vascular damage, the most serious one is injury of the internal carotid artery (ICA), which leads to disastrous consequences if not treated properly (4). An uncommon but serious vascular complication of EETS, which typically arises due to intraoperative injury of the sphenopalatine artery and its branches (5–8). Inadequate management of postoperative epistaxis can lead to serious consequences, such as asphyxia and hypovolemia, even hemorrhagic shock and death (9, 10). Nevertheless, it has been reported that bleeding sites cannot be identified in some cases, the effect of nasal packing is not completely satisfactory. In this study, we retrospectively analyzed 8 consecutive cases of epistaxis following EETS, investigating the incidence, risk factors, management and prevention of post-operative epistaxis.

## Patients and methods

### Patients and surgical techniques

All patients with sellar lesions such as PA, Rathke's cleft cyst, craniopharyngioma, clival chordoma or cerebral fluid leakage who underwent EETS in Beijing Xuanwu Hospital, one of the largest tertiary referral centers in China between September 2020 and November 2022 were retrospectively analyzed. All the patients included had complete clinical, radiological, and biochemical data, were treated with EETS and had at least 6 months of follow-up.

All EETS procedures were performed by authors Liu X and Ge Chen using a Karl Storz endoscope through a binostril approach. After general anesthesia and orotracheal intubation, the patients were placed in supine position with the upper body elevated 20 degrees and the head turned 10 degrees toward the surgeon. Both nostrils were washed with a topical vasoconstrictor. After the preparation of nasal cavity using adrenaline-soaked cotton, the middle turbinate was first pushed laterally to facilitate the introduction of instruments. A wide sphenoidotomy was done using Kerrison Rongeurs and high-speed diamond drills and the sellar floor, clivus, carotid prominences, opticocarotid recesses and planum sphenoidale were exposed. After tumor resection and hemostasis, the skull base was reconstructed based on the anatomical level. For patients received extended EETS procedures with high-flow postoperative cerebrospinal fluid rhinorrhoea, multilayer regimes were utilized with fat grafts, fascia lata grafts and Hadad–Bassagasteguy vascularized septal flaps. For cases with no perioperative CSF leak, only Gel foam and fibrin glue were used to close the sella. The nasal cavity was packed with iodoform gauzes for additional support to the skull base reconstruction if necessary, which was removed after 3–21 days.

Postoperative epistaxis was defined as severe, persistent or recurrent arterial nosebleed which required therapeutic intervention. Patients with limited nasal bleeding that required no treatment were excluded. Patients who developed significant or recurrent epistaxis requiring therapeutic intervention and readmission to the hospital, either immediately after transnasal transsphenoidal surgery or, up to 30 days postoperatively were included. All the following data were collected: sex, age, pathology, postoperative time course of epistaxis occurrence, epistaxis inducements, bleeding arteries, managements, complications sand follow-up time. The study was approved by the Research Ethics Committee of Beijing Xuanwu Hospital, and informed consent was obtained from all patients.

### Treatment and follow-up

According to the standard procedure for postoperative epistaxis in our center, nasal packing was performed for all patients as soon as they arrived at the emergency room (ER), and intensive care was given. If the nasal packing was insufficient and the nose bleeding continued, the patients were taken to the operation room (OR) and treated with endoscopic bleeding artery electrocauterization under general anesthesia with endotracheal intubation. All procedures were performed by a single surgeon (X. Liu). The patient was placed in a supine position with the head elevated by 30 degree. First, blood clots in the nasal cavity were evacuated, followed by a thorough endoscopic examination of both nasal cavities to search for the bleeding site. Once the bleeding artery was identified, it was electrocoagulated with bipolar diathermy. Other suspicious bleeding points and the mucosal cut edge were also coagulated. After completion of hemostasis, iodoform gauze was used for nasal packing, which was removed under endoscopic view after 3–7 days. Patients were followed up for 6–22 months with particular attention given for any further epistaxis.

## Results

### Patient characteristics

A total of 557 patients with sellar lesions such as PA, Rathke's cleft cyst, craniopharyngioma or clival chordoma who underwent EETS in Beijing Xuanwu Hospital between September 2020 and November 2022 were retrospectively analyzed. Among them, eight (1.4%) cases were identified as having postoperative epistaxis, including 4 males and 4 females, with a median age of 47.12 years (27–70 years). Pathological results of the EETS consisted of 8 PAs (2 somatotroph adenomas, 2 plurihormonal PIT-1–positive adenomas, 1 thyrotroph adenoma 1 silent corticotroph adenoma and 1 gonadotroph adenoma) and 1 Rathke's cleft cyst. The size of the PAs was 9.6 mm–46.2 mm, with a median size of 22.1 mm. Epistaxis occurred 4 h to 30 days after EETS, with a median of 14.5 days (Table 1). Coagulation function test in all the 8 patients were normal. Five in 7 patients with PAs were Knosp grade 3 while the other two were grade 1 or 2. The inducing factors for epistaxis included administration of antiplatelet agents (ticagrelor plus aspirin) in 1 patient one week after operation, nose blowing in 2 patients and removal of nasal packing in 1 patient. One patient was infected with COVID-19 during the pandemic, and the epistaxis occurred 30 days postoperatively during the upper respiratory tract infection, including nasal dryness and cough, which could be the potential cause of the nose bleeding. Among the remaining 3 patients, the risk factors were still unknown. After careful review of the surgical videos, we found all 8 patients had downward extension of the septal mucosal incision, in which 6 had intraoperative bleeding of posterior nasal septal artery (PNSA) that were cauterized by bipolar diathermy. Detailed clinical characteristics of all patients are shown in Table 1.

**Table 1:** Detailed characteristics of the 8 patients with postoperative epistaxis.

No.	Sex	Age (y)	Tumor size (mm)	Knosp grade	Pituitary function	Pathology	Approach	Surgical results	Postoperative time of epistaxis occurrence	Bleeding inducements	Bleeding arteries	Treatment	Follow-up time (m)
1	Female	27	24.4	2	Adrenal deficient	Somatotroph adenoma	Standard	GTR	8 days	Nose blowing	–	Anterior nasal packing	29
2	Male	49	18.0	3A in the right	Thyroid hypofunction	Plurihormonal PIT-1-postive adenoma	Standard	GTR	17 days	Administration of antiplatelet agent	–	Posterior nasal packing	24
3	Female	70	19.8	3B in the left	Normal	Corticotroph adenoma	Standard	GTR	29 days	–	Left PNSA	Posterior nasal packing followed by bleeding artery cauterization	24
4	Male	52	44.2	3B in the right	Normal	Gonadotroph adenoma	Standard	GTR	4 h	–	Right PNSA	Anterior nasal packing followed by bleeding artery cauterization	13
5	Male	48	9.6	1	Normal	Thyrotroph adenoma	Standard	GTR	21 days	Nose blowing	–	Posterior nasal packing	12
6	Male	43	10.2	1	Panhypopituitarism	Rathke's cleft cyst	Standard	GTR	5 h	–	Left PNSA	Anterior nasal packing followed by bleeding artery cauterization	11
7	Female	34	24.5	3B in the left	Normal	Plurihormonal PIT-1-postive adenoma	Standard	GTR	9 days	Removal of nasal packing	Left PNSA	Posterior nasal packing followed by bleeding artery cauterization	10
8	Female	54	26.4	3A in the left	Normal	Somatotroph adenoma	Standard	GTR	30 days	Covid-19 infection and nose blowing	Left PNSA	Posterior nasal packing followed by bleeding artery cauterization	2

### Treatment and follow-up

Intermittent nasal packing was performed in all 8 patients as soon as they arrived at the ER, and the nose bleeding was successfully stopped in 3 patients. The remaining 5 patients were taken to the OR and PNSA was defined as the responsibility vessel (4 left-sided/1 right-sided). Four in 5 patents identified with responsible blood vessels were Knosp 3 grade at the same direction. The bleeding artery was not clearly identified in the other 3 patients, whose bleeding was stopped after nasal packing. No patients experienced rebleeding during the follow-up period (median 15.6 months).

## Discussion

In addition to intraoperative ICA injury, postoperative epistaxis is another uncommon but severe vascular complication of EETS, with an incidence of 0.6%–3.3% (5, 6, 11–15). According to a retrospective cross-sectional analysis of a total of 5,891 adult patients with pituitary adenoma treated via endoscopic transsphenoidal approach using the Nationwide Readmissions Database between 2010 and 2015, the risk of postoperative epistaxis was 0.71% (16). Without intermittent and proper treatment, post-EETS epistaxis may result in several complications, leading to serious consequences, such as asphyxia and hypovolemia, even ischemic shock and death, not to mention a relatively high medical expense (17). The risk factors for post-EETS have not been well investigated and management for epistaxis would be various, including nasal packing, electrocauterization of the bleeding artery and endovascular embolization (18). When the effect of nasal packing is not always completely satisfying, especially for active arterial bleeding, patients are taken to the OR and treated with endoscopic bleeding artery electrocauterization or endovascular embolization (19). Here, we reported 8 cases of epistaxis following EETS, emphasizing the incidence, risk factors, management and prevention of the condition in our center.

**Figure 1:**
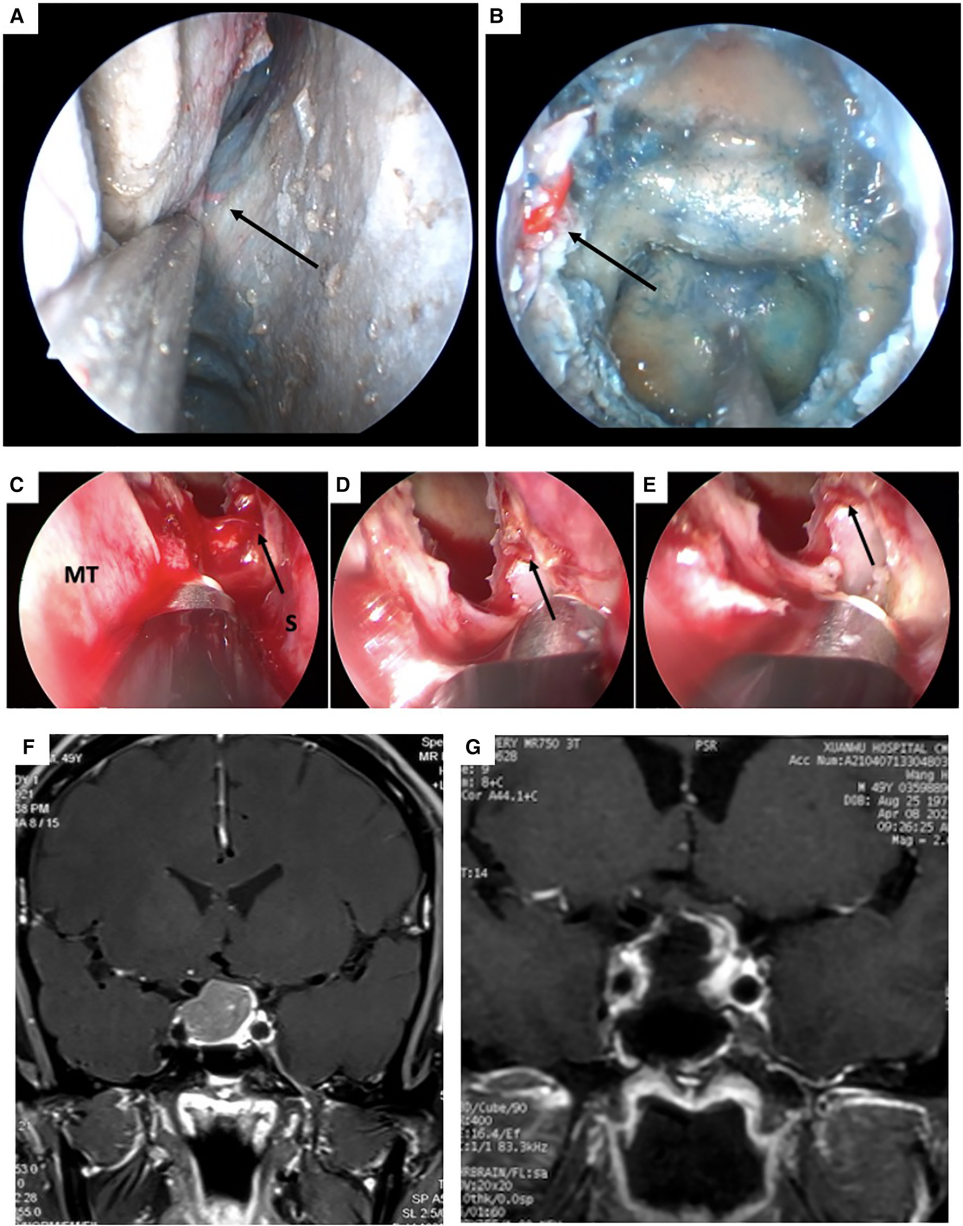
Cadaveric view of the right posterior nasal septal artery (PNSA) and intraoperative view of the bleeding originated from the left PNSA of case 7. (**A**) The right PNSA. (**B**) The right PNSA after opening the sphenoidal sinus. (**C**) The left PNSA was injured and identified as the bleeding source (white arrow). (**D**) The PNSA was cauterized with a bipolar electrocoagulation forceps. (**E**) Hemostasis was successfully achieved. (**F** and **G**) pre-and post-operation MRI scan of case 7. PNSA: posterior nasal septal artery; MT: middle turbinate; S: septum.

In our series, the incidence of post-EETS was 1.4%, which is relatively similar to that in previous reports (5, 6, 11–15). Intraoperative injury of the sphenopalatine artery and its branches, including direct injury and indirect retraction injury, is an important cause of epistaxis following EETA (5, 6, 19–21). Other vessels, including the ICA and posterior ethmoidal artery, have only been sporadically reported (22, 23). The sphenopalatine artery exits from the sphenopalatine foramen and supplies the majority of the nasal mucosa; its two major branches are the lateral nasal septal artery and PNSA (Figures 1A,B). The PNSA runs along the anteroinferior wall of the sphenoid sinus and is distributed on the posterior portion of the nasal septum (24). In EETS, PNSA injury usually occurs when the sphenoidal ostium is enlarged in an inferior direction, which is regarded as an important risk factor for epistaxis (25). In the current series, the same definitive bleeding source, the PNSA, was found in 5 of 8 patients. After careful review of the obvious surgical videos, we found all 8 patients had downward extension of the septal mucosal incision, in which 6 had intraoperative bleeding of PNSA that were cauterized by bipolar diathermy. In our case series, the mucosa was incised with a cryogenic plasma ablation probe, which also produces simultaneous hemostasis (Figure 1C). Therefore, the PNSA could be temporarily occluded during the incision phase of the operation (Figure 1D). The pre- and post-operative MR scan of the PA in case #2 were shown in Figures 1F,G. However, the occlusion of the PNSA may have been incomplete and less target than bipolar cauterization, which is a potential risk factor for postoperative epistaxis (Figure 1E). For the 3 patients in whom the responsible artery was not identified, the possibility of PNSA injury could not be excluded. The 8 patients with epistaxis consisted of 7 with PAs and 1 with a Rathke's cyst, for whom standard EETS instead of extended EETS was adopted. However, four in 5 patents identified with responsible blood vessels were Knosp 3 grade at the same direction, in which the septum incision was extended inferiorly down and could easily injured PNSA.

Kenneth et al. suggested that direct intraoperative arterial injury was the cause of immediate postoperative epistaxis, while no definite cause of delayed postoperative epistaxis was identified (5). In our series, 2 of the 8 patients developed epistaxis on the day of surgery. Although there were no obvious causative factors in these 2 patients, endotracheal extubation-induced cough and Valsalva maneuvers have been considered to be inducements of immediate postoperative epistaxis, as they may cause the rupture of the unreliably coagulated PNSA and ultimately epistaxis. For the 6 patients who had epistaxis 2 days postoperatively, the causes were still obscure. According to Alzhrani et al., most delayed epistaxis occurs 1–3 weeks after surgery (26). Wang et al. reported that the potential causes for epistaxis were present at all times within 10 weeks postoperation (15). During this time period, a series of pathophysiological responses are activated, including scab dissolution, mucosal edema, fibrous connective tissue hyperplasia, and vascular remodeling in the operation area, associated with an increased bleeding propensity (15). The peak period for the onset of epistaxis is 6–20 days after surgery (15). In our study, out of the 8 patients included, 6 had delayed epistaxis. One patient developed epistaxis immediately after the iodoform gauze packing strips were removed on the 9th postoperative day, and the other patient developed epistaxis on the 29th postoperative day with no obvious causative factors. One patient was infected with COVID-19, and epistaxis occurred 40 days postoperatively during the severe upper respiratory tract infection, including headache, nasal dryness and cough, which could be the potential cause of nose bleeding.

The site of bleeding was undefined in 3 of the 8 patients, whose epistaxis was successfully treated with nasal packing. The epistaxis was preceded by an administration of antiplatelet agents (ticagrelor plus aspirin) in one patient one week after operation and a Valsalva-like maneuver or nose blowing in two patients. All the above factors are considered to induce epistaxis. Other risk factors have also been reported, including secondary surgery, use of a nasoseptal flap, pseudoaneurysm, and local infection (8, 23, 27, 28), which did not occur in our case series.

Once active epistaxis is confirmed, routine anterior nasal packing is ineffective (8, 15). Because the bleeding source is usually the sphenopalatine artery or its branches located in the posterior nasal cavity. In contrast, epistaxis can likely be stopped by endoscopic posterior nasal packing in some cases. However, it often reoccurred after removal of the packing. For early postoperative epistaxis that had not been successfully treated with nasal packing, Kenneth et al. believed the patient should be taken to the OR for endoscopic exploration to determine the source of bleeding so the bleeding artery would be relatively easy to be identified (5).

In our present series, all 8 patients received endoscopic posterior nasal packing and epistaxis in 3 patients was stopped, while the nasal packing was not effective in remaining 5 patients. So, endoscopic posterior nasal packing may not be a reliable option for active epistaxis. For an endoscopic exploration under local anesthesia, many patients cannot tolerate the surgery due to fear and pain and a thorough endoscopic examination of both nasal cavities to search for the bleeding site was not satisfying. Furthermore, this can also lead to an acute increase in blood pressure and exacerbate bleeding, making it much more difficult to search for the bleeding source. If hemostasis cannot be successfully achieved in a short period of time, the blood may lead to airway obstruction and asphyxia without endotracheal intubation. In contrast, these problems could be avoided under general anesthesia, and there's plenty of time to find the bleeding artery and achieve successful hemostasis (29). During endoscopic exploration of our 5 patients who required a second operation, there are numerous blood clots in the nasal cavity and the nasal mucosa was congested and edematous. After the blood clots were cleared carefully, the bleeding artery was identified in all 5 patients, which was very difficult to achieve under local anesthesia.

Endovascular embolization has been considered an effective treatment for epistaxis (5, 7, 8, 17, 21). However, angiography often fails to find the bleeding artery, and empiric maxillary arterial embolization is needed, which is associated with some complications, such as cerebrovascular accident, hemiplegia, ophthalmoplegia, facioplegia, epileptic seizure, and soft tissue necrosis (17, 30). In addition, endovascular embolization is more expensive than other treatments. Bleeding artery electrocauterization can achieve more precise hemostasis and thus greatly attenuate complications due to occlusion of the maxillary artery. Hence, for epistaxis that is refractory to nasal packing, we believe that endoscopic bleeding artery electrocauterization should be immediately performed under general anesthesia in the operating room, regardless of the timing of the epistaxis. In addition, although not seen in the current series, the bleeding artery may still be difficult to identify even under general anesthesia in some cases. Under such conditions, endoscopic sphenopalatine artery ligation appears to be a better option. In comparison with endovascular embolization, endoscopic sphenopalatine artery ligation has been reported to have a comparable effect on epistaxis with fewer complications and lower costs (31, 32).

Avoidance of intraoperative vascular injury is essential for preventing postoperative epistaxis. During EETS, extended sphenoidotomies are frequently required for sufficient exposure and total resection of tumors. The PNSA crosses the lower portion of the anterior wall of the sphenoid sinus and is located in the mucosal layer (24). In our series, all 8 patients had downward extension of the septal mucosal incision, in which 6 had intraoperative bleeding of PNSA that were cauterized by bipolar diathermy. Consequently, it is recommended that the mucosa should be separated from the bony wall and turned downward to obtain adequate exposure for tumor resection while avoiding PNSA injury (6, 24).

**Figure 2:**
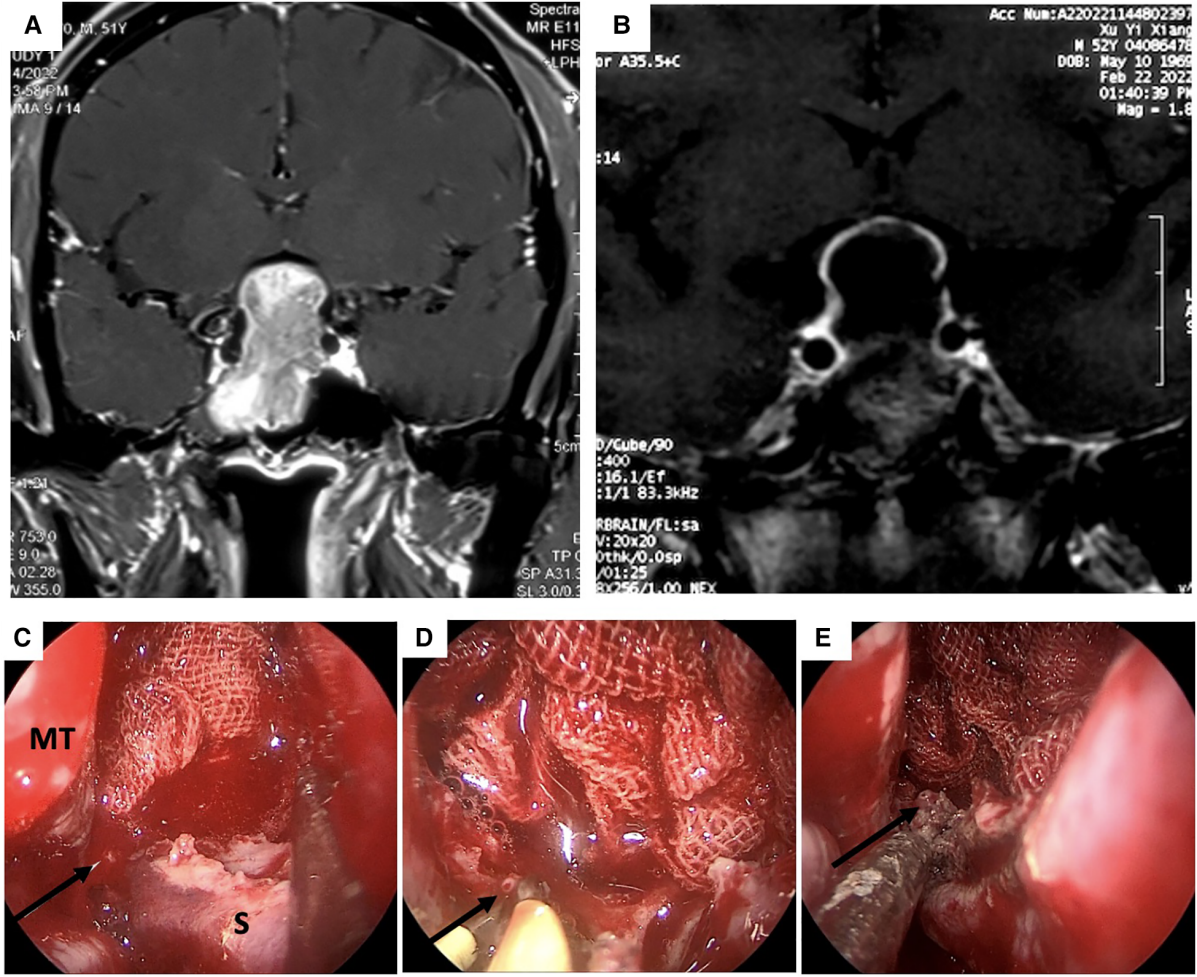
MRI scan and intraoperative view of the epistaxis originated from the right posterior septal artery (PNSA) of case 4. (**A** and **B**) pre-and post-operation MRI scan of case 4 (**C**) The right PNSA was identified as the bleeding source. (**D**) The PNSA was clamped and cauterized with a cryogenic plasma ablation probe. (**E**) Hemostasis was successfully achieved. PNSA: posterior nasal septal artery; MT: middle turbinate.

In our cases, intraoperative arterial bleeding was controlled with a cryogenic plasma ablation probe, which while highly effective and safe has certain shortcomings as well. The tip of the probe is relatively wide, so it may be difficult to establish hemostasis for arteries in crevices or rugged surfaces. In contrast, a fine-tipped bipolar electrocoagulation forceps can accurately clamp the bleeding artery and thoroughly cauterize it (Figures 2A–C). The pre- and post-operative MR scan of the PA in case #4 were shown in Figures 2D,E. To the best of our knowledge, no previous studies have compared the hemostatic efficacy of cryogenic plasma ablation to bipolar electrocoagulation in EETA. Nevertheless, our experience suggests that the combination of the two devices could provide better hemostatic effects by leveraging the advantages of both.

## Conclusion

PNSA is the most common bleeding source of sever postoperative epistaxis following EETA. To prevent postoperative epistaxis, the incision of the septal mucosa should not be extended downward excessively, and intraoperative hemostasis should be ensured by bipolar electrocoagulation. Once active epistaxis is confirmed, the effect of nasal packing is less favorable, while endoscopic bleeding artery electrocauterization under general anesthesia may be a safe and effective measure.

## Data Availability

The original contributions presented in the study are included in the article, further inquiries can be directed to the corresponding author.

## References

[1] Jankowski R Auque J Simon C Hepner H Wayoff M. Endoscopic pituitary tumor surgery. Laryngoscope. (1992) 102(2):198–202. 10.1288/00005537-199202000-00016 1738293

[2] Henderson F Jr Schwartz TH. Update on management of craniopharyngiomas. J Neurooncol. (2022) 156(1):97–108. 10.1007/s11060-021-03906-4 34807341

[3] Vignolles-Jeong J Kreatsoulas D Godil S Otto B Carrau R Prevedello D. Complications in endoscopic pituitary surgery. Otolaryngol Clin North Am. (2022) 55(2):431–48. 10.1016/j.otc.2021.12.011 35365316

[4] Sharma RK Irace AL Overdevest JB Gudis DA. Carotid artery injury in endoscopic endonasal surgery: risk factors, prevention, and management. World J Otorhinolaryngol Head Neck Surg. (2022) 8(1):54–60. 10.1002/wjo2.7 35619937 PMC9126167

[5] Kenneth M Gross BA Frerichs KU Ian FD Lin N Rincon-Torroella J Incidence, risk factors and management of severe post-transsphenoidal epistaxis. J Clin Neurosci. (2015) 22(1):116–22. 10.1016/j.jocn.2014.07.004 25150759

[6] Thompson CF Wang MB Kim BJ Bergsneider M Jeffrey DS. Incidence and management of epistaxis after endoscopic skull base surgery. Orl. (2012) 74(6):315–9. 10.1159/000345500 23258350

[7] Nishioka H Ohno S Ikeda Y Ohashi T Haraoka J. Delayed massive epistaxis following endonasal transsphenoidal surgery. Acta Neurochir. (2007) 149(5):523–7. 10.1007/s00701-007-1134-0 17404684

[8] Cockroft KM Carew JF Trost D Fraser R. Delayed epistaxis resulting from external carotid artery injury requiring embolization: a rare complication of transsphenoidal surgery: case report. Neurosurgery. (2000) 47(1):236–9. 10.1097/00006123-200007000-00052 10917369

[9] Wang L Wang X Ba Y. Clinical analysis of delayed epistaxis following endoscopic sinus surgery. Am J Otolaryngol. (2022) 43(3):103406. 10.1016/j.amjoto.2022.103406 35378344

[10] Traboulsi H Alam E Hadi U. Changing trends in the management of epistaxis. Int J Otolaryngol. (2015) 20(15):263–987. 10.1155/2015/263987 PMC4553192 26351457

[11] Siedek V Pilzweger E Betz C Leunig A. Complications in endonasal sinus surgery: a 5-year retrospective study of 2,596 patients. Eur Arch Oto-Rhino-Laryngol. (2013) 270(1):141–8. 10.1007/s00405-012-1973-z 22466016

[12] Stankiewicz JA Lal D Connor M Welch K. Complications in endoscopic sinus surgery for chronic rhinosinusitis: a 25-year experience. Laryngoscope. (2011) 121(12):2684–701. 10.1002/lary.21446 22086769

[13] Cavallo LM Briganti F Cappabianca P Maiuri F Valente V Tortora F Hemorrhagic Vascular Complications of Endoscopic Transsphenoidal Surgery. min-Minim Invasive Neurosurgery. (2004) 47(03):145–50. 10.1055/s-2004-818489 15343429

[14] Cappabianca P Cavallo LM Colao AM Divitiis E. Surgical complications associated with the endoscopic endonasal transsphenoidal approach for pituitary adenomas. J Neurosurg. (2002) 97(2):293–8. 10.3171/jns.2002.97.2.0293 12186456

[15] Rudmik L Leung R. Cost-effectiveness analysis of endoscopic sphenopalatine artery ligation vs arterial embolization for intractable epistaxis. JAMA Otolaryngology–Head & Neck Surgery. (2014) 140(9):802–8. 25123233 10.1001/jamaoto.2014.1450

[16] Al-Qurayshi Z Bennion DM Greenlee JDW Graham SM. Endoscopic pituitary surgery: national database review. Head Neck. (2022) 44(12):2678–85. 10.1002/hed.27179 36039744

[17] He W Shen M Chen Z Ma Z Qiao N Zhang Y. Risk factors of epistaxis after endoscopic endonasal skull base surgeries. Clin Neurol Neurosurg. (2022) 217:107243. 35487040 10.1016/j.clineuro.2022.107243

[18] Willems PWA Farb RI Agid R. Endovascular treatment of epistaxis. Am J Neuroradiol. (2009) 30(9):1637–45. 10.3174/ajnr.A1607 19372207 PMC7051515

[19] Tunkel DE Anne S Payne SC IshmanLS L Rosenfeld RMM Abramson PJ Clinical practice guideline: nosebleed (epistaxis). Otolaryngol Head Neck Surg. (2020) 162(1_suppl):S1–38. 31910111 10.1177/0194599819890327

[20] Sylvester PT Moran CJ Derdeyn CP Cross DT Dacey RG Zipfel GJ Endovascular management of internal carotid artery injuries secondary to endonasal surgery: case series and review of the literature. J Neurosurg. (2016) 125(5):1256–76. 10.3171/2015.6.JNS142483 26771847

[21] Ramachandran SN Retnathankom A Rege I Reddy KT. Endovascular management of a sphenopalatine artery pseudoaneurysm: a rare cause of delayed intractable epistaxis following endoscopic transsphenoidal pituitary surgery. BMJ Case Rep. (2023) 16(5):e253998. 37130637 10.1136/bcr-2022-253998 PMC10163417

[22] Raymond J Hardy J Czepko R Roy D. Arterial injuries in transsphenoidal surgery for pituitary adenoma; the role of angiography and endovascular treatment. Am J Neuroradiol. (1997) 18(4):655–65. 9127026 PMC8338477

[23] Hayashi Y Daisuke K Iwato M Nakanishi S Hamada J. Massive hemorrhage from the posterior ethmoidal artery during transsphenoidal surgery: report of 2 cases. Turk Neurosurg. (2015) 25(5):804–7. 26442552 10.5137/1019-5149.JTN.11013-14.0

[24] Kepron C Cusimano M Pollanen MS. Fatal hemorrhage following trans-sphenoidal resection of a pituitary adenoma: a case report and review of the literature. Forensic Sci Med Pathol. (2010) 6(4):282–7. 10.1007/s12024-010-9154-0 20306333

[25] Lee HY Kim HU Kim SS Son EJ Kim JW Cho NH Surgical anatomy of the sphenopalatine artery in lateral nasal wall. Laryngoscope. (2002) 112(10):1813–8. 10.1097/00005537-200210000-00020 12368621

[26] Kam J Ahmad A Williams A Peterson E Craig J. Postoperative epistaxis and sphenoid sinus ostial stenosis after posterior septal branch injury during sphenoidotomy. Int Forum Allergy Rhinol. (2019) 9(8):842–9. 31012265 10.1002/alr.22345

[27] Alzhrani G Sivakumar W Park MS Taussky P Couldwell W. Delayed complications after transsphenoidal surgery for pituitary adenomas. World Neurosurg. (2018) 109:233–41. 10.1016/j.wneu.2017.09.192 28989047

[28] Hadad G Bassagasteguy L Carrau RL Mataza J Kassam A Snyderman C A novel reconstructive technique after endoscopic expanded endonasal approaches: vascular pedicle nasoseptal flap. Laryngoscope. (2006) 116(10):1882–6. 10.1097/01.mlg.0000234933.37779.e4 17003708

[29] Campbell RG. Sphenopalatine artery pseudoaneurysm after endoscopic sinus surgery: a case report and literature review. Ear Nose Throat J. (2012) 91(2):E4–11. 10.1177/014556131209100215 22359145

[30] McClurg SW Carrau R. Endoscopic management of posterior epistaxis: a review. Acta Otorhinolaryngol Ital. (2014) 34(1):1. 24711676 PMC3970224

[31] Koh E Frazzini VI Kagetsu NJ. Epistaxis: vascular anatomy, origins, and endovascular treatment. Am J Roentgenol. (2000) 174(3):845–51. 10.2214/ajr.174.3.1740845 10701637

[32] De Bonnecaze G Gallois Y Bonneville F Vergez S Chaput B Serrano E. Transnasal endoscopic sphenopalatine artery ligation compared with embolization for intractable epistaxis: a long-term analysis. Am J Rhinol Allergy. (2018) 32(3):188–93. 10.1177/1945892418768584 29676168

